# Ultrafast Dynamics of Demagnetization in FeMn/MnGa Bilayer Nanofilm Structures via Phonon Transport

**DOI:** 10.3390/nano12224088

**Published:** 2022-11-20

**Authors:** Tianran Jiang, Xupeng Zhao, Zhifeng Chen, Yongyong You, Tianshu Lai, Jianhua Zhao

**Affiliations:** 1 State Key Laboratory of Optoelectronic Materials and Technologies, School of Physics, Sun Yat-Sen University, Guangzhou 510275, China; 2 State Key Laboratory of Superlattices and Microstructures, Institute of Semiconductors, Chinese Academy of Sciences, P.O. Box 912, Beijing 100083, China; 3 School of Physics and Electronic Engineering, Guangzhou University, Guangzhou 510006, China

**Keywords:** antiferromagnet/ferromagnet, nanofilm structures, phonon transport, ultrafast demagnetization, time-resolved magneto-optical Kerr effect

## Abstract

Superdiffusive spin transport has been proposed as a new mechanism of ultrafast demagnetization in layered magnetic nanostructures and demonstrated experimentally. However, it is unknown if it is possible for phonon transport to occur and manipulate ultrafast demagnetization. Here, we explore the ultrafast dynamics of demagnetization of an antiferromagnet/ferromagnet bilayer nanostructure, of a FeMn/MnGa bilayer film prepared by molecular beam epitaxy. Ultrafast dynamics of a two-step demagnetization were observed through the time-resolved magneto-optical Kerr effect. The first-step fast component of the two-step demagnetization occurred within ~200 fs, while the second-step slow component emerged in a few tens of picoseconds. For a single MnGa film, only the ultrafast dynamics of the first-step fast demagnetization were observed, revealing that the second-step slow demagnetization originates from interlayer phonon transport. A four-temperature model considering phonon transport was developed and used to effectively reproduce the observed ultrafast dynamics of two-step demagnetization. Our results reveal the effect of phonon transport on demagnetization for the first time and open up a new route to manipulate ultrafast demagnetization in layered magnetic structures.

## 1. Introduction

Since a laser-induced ultrafast demagnetization was first demonstrated in a pioneering experiment on single Ni film [1], many studies have been carried out on different magnetic materials through a time-resolved magneto-optical Kerr effect (TR-MOKE), including FePt [2], CoPt [3], CrO_2_ [4], GdFeCo [5], Co_2_FeAl [6] and TbFeCo [7,8]. The laser-induced demagnetization is usually described by a three-temperature model (3T-M) in which the temperatures of electron, spin and lattice subsystems coupled to each other and their time evolution was described by a set of derivative equations, while the time evolution of the temperature of the spin subsystem described the ultrafast dynamics of magnetization after photon excitation [1,9,10]. However, the microscopic mechanisms of ultrafast demagnetization on femtosecond timescales are still being strongly debated. Several different mechanisms were presented, such as a direct coupling between light field and spin bath [11], coulomb-exchange spin flips [12] and phonon-mediated spin-flip processes [13], etc.

In recent years, a new superdiffusive spin transport mechanism was proposed to explain the ultrafast demagnetization process in layered magnetic structures [14], and has already been demonstrated successfully in ferromagnetic/nonmagnetic (FM/NM) [15,16,17,18] and ferromagnetic/ferromagnetic (FM/FM) [19,20] layered structures. Based on 3T-M, each FM layer contained three subsystems of electrons, spins and lattices, whereas an NM layer had only electron and lattice subsystems. Consequently, there might be other transports occurring between different layers in layered magnetic structures besides a superdiffusive spin transport, such as hot electron transports and phonon transports. In fact, ultrafast demagnetization driven by a hot electron transport from an NM layer into an FM layer has already been observed in an NM/FM layered magnetic structure [21,22], where the NM layer was an Au [21] or Cu film [22], while the FM layer was a Ni [21] or [Co/Pt]_n_ [22] film. However, the ultrafast dynamics of demagnetization driven by phonon transports have not been observed and reported to our knowledge. Phonon transports may also open up a new route to manipulate ultrafast demagnetization in a layered magnetic structure, and thus are critical to explore. To observe the effect of phonon transports on ultrafast demagnetization, it is essential to suppress the effect of hot electron and spin transports. Therefore, the design of layered magnetic structure samples becomes very important and is the key factor which affects whether the phonon transport is observable or not.

In this paper, we designed an antiferromagnetic/ferromagnetic (AFM/FM) bilayer nanostructure, using FeMn/MnGa grown on a GaAs substrate and studied its ultrafast dynamics of demagnetization to attempt the observation of possible phonon transports. Here, the AFM layer of FeMn was chosen because Mn-based AFM materials, such as FeMn, PdMn, IrMn and PtMn, have an extremely short electron and spin diffusion length on the order of 1 nm [23,24,25] and have zero net magnetizations; thus, hot electron and spin transports are significantly suppressed. On the other hand, it was reported that the exchange bias field between the FeMn and MnGa layers disappeared at room temperature [26], thus avoiding a possible laser-induced magnetization change in the MnGa layer driven by the exchange bias field. Consequently, the ultrafast dynamics of demagnetization in an MnGa single layer could be observed after photoexcitations. We studied the ultrafast dynamics of demagnetization of FeMn/MnGa bilayer and MnGa single layer films comparatively using TR-MOKE. We indeed observed the ultrafast dynamics of two-step demagnetization in an FeMn/MnGa layered structure, while only the ultrafast dynamics of one-step demagnetization were observed in a single layer MnGa film. The second-step slower ultrafast demagnetization appeared only in FeMn/MnGa bilayer structure and had a time constant of ~10 ps, and was thus ascribed to the phonon transport from the FeMn layer because electron and spin transports were suppressed in this sample structure and usually took place within a subpicosecond time scale. We developed a four-temperature model by introducing the temperature of the phonon subsystem of an FeMn layer into the 3T-M and taking account for its coupling to the temperatures of electron, spin and phonon subsystems of the MnGa layers. Simulation calculations were performed based on the four-temperature model. The simulated results closely reproduced our experimental results of the ultrafast dynamics of two-step demagnetization, further confirming the origin of phonon transports of the second-step slower demagnetization. Therefore, we observed for the first time the effect of phonon transports on ultrafast demagnetization in a bilayer magnetic nanostructure.

## 2. Materials and Experimental Method

FeMn/MnGa bilayer films were deposited on GaAs film substrates by molecular-beam epitaxy (MBE), as described in detail in reference [26]. It was expected that an MBE-prepared sample could lead to a good interlayer phonon transport because a lattice match was required in MBE, so that almost no phonon scattering would occur at the magnetic interface of the FeMn/MnGa heterostructure. The sample structure consisted of FeMn (3 nm)/MnGa (8 nm)/GaAs (200 nm). Magnetic moments of MnGa film were dominated by only 3*d* electrons near the Fermi level [27], whereas FeMn was antiferromagnetic and had no net moments because the paired Fe and Mn atoms were arranged anti-parallel in the FeMn films, respectively [23,24].

The ultrafast dynamics of demagnetization were studied through TR-MOKE measurement. A laser pulse with a central wavelength of 800 nm, a duration of ~100 fs and a repetition rate of 1 kHz was generated from a Ti: sapphire regenerative amplifier. The laser pulse was divided into strong pump and weak probe with a pump/probe intensity ratio larger than 20. The pump and probe pulses were incident on the sample almost normally, and focused on a spot of 150 μm and 75 μm for the pump and the probe, respectively. The polar Kerr rotation of the reflected probe light was detected by an optical balanced bridge and was measured by a lock-in amplifier synchronized to an optical chopper that modulated pump pulses at ~340 Hz. All measurements were performed at room temperature.

## 3. Results and Discussion

**Figure 1:**
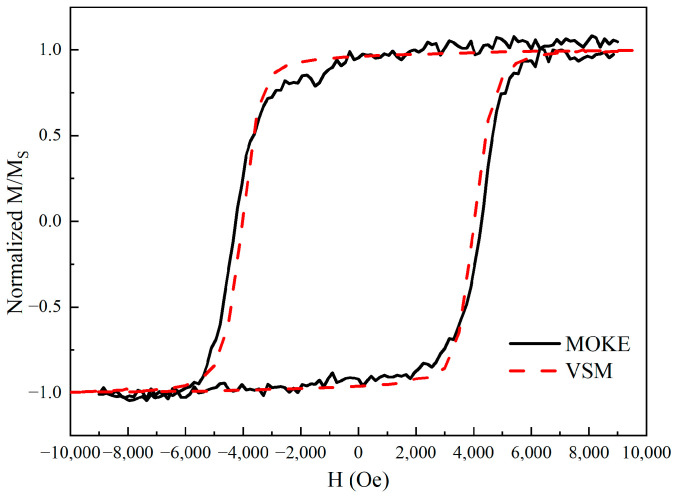
Measurement of the normalized out-of-plane hysteresis loops. The dash and solid loops are measured by VSM and MOKE, respectively.

The magnetic properties of the sample were first characterized by polar MOKE and a vibrating sample magnetometer (VSM) with an external field applied perpendicularly to the surface of the sample. As shown in Figure 1, the normalized out-of-plane hysteresis loops present nearly square and centric-symmetric, and show a coercivity of ~4500 Oe, which reveal that this sample has a strong perpendicular magnetic anisotropy and a zero exchange bias field between the FeMn and MnGa layers at room temperature, consistent with a previous report [26]. The square out-of-plane hysteresis loops agree well with the sole MnGa film reported previously [27], revealing the completely antiferromagnetic property of FeMn. Therefore, the magnetic signal measured by MOKE and VSM originates from the sole MnGa layer, excluding the FeMn layer. In this way, we can study the time evolution of magnetization in the sole MnGa layer after the photoexcitation of FeMn/MnGa bilayered structures.

**Figure 2:**
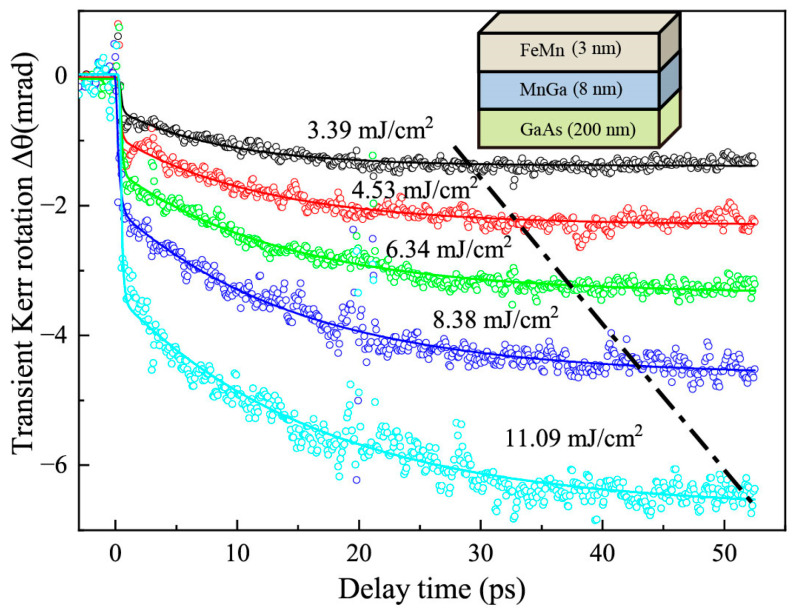
The pump–fluence dependence of TR-MOKE measurement in an FeMn/MnGa layered structure. The scattered open circles are Kerr signal measurements. The solid lines are the fittings to experimental profiles with a double exponential sum function described in the text (Equation (1)). The dot-dashed line joins the maximum demagnetization points at different pump fluence levels.

The ultrafast dynamics of laser-induced demagnetization of the FeMn/MnGa layered structure was measured experimentally using TR-MOKE spectroscopy for different pump fluence levels under a saturation magnetic field of ~8000 Oe applied along the normal of the sample plane, and plotted in Figure 2. One can see that the ultrafast dynamics present a two-step ultrafast demagnetization process, including a fast demagnetization process occurring within a picosecond and a slower process in several tens of picoseconds, as the dot-dashed line shows. The amplitude of the two demagnetization processes increases with pump fluence. The fast demagnetization process is easily understood based on 3T-M [1] and can be ascribed to electron–spin coupling in the MnGa film layer. A similar fast demagnetization process was observed in many FM films, such as Ni [1], FePt [2] and CoPt [3]. However, the slower demagnetization process was not observed usually [1,2,3]. Here, there are two possible origins for the slower demagnetization process. One is an intralayer phonon–spin coupling interaction that re-heats spins in the MnGa layer based on 3T-M. Similar phenomena were observed in CrO_2_ [4], Fe_3_O_4_ [28] and Mn_2_Ru_x_Ga [29]. The other is interlayer transport. Electron and spin transports can be ruled out because they occurred in a subpicosecond time scale [15,17,19,20,22,30], whereas here the second-step slower demagnetization emerges over 10 ps. On the other hand, the FeMn layer is antiferromagnetic, and has an extremely short electron and spin diffusion length of ~1 nm [23,24,25], suppressing electron and spin transports into the MnGa layer from the FeMn layer. As a result, only phonon transport becomes possible. Similar interlayer phonon transports were reported in a nonmagnetic bilayer structure of Pt/Au and indeed occurred in several tens of picoseconds [31].

**Figure 3:**
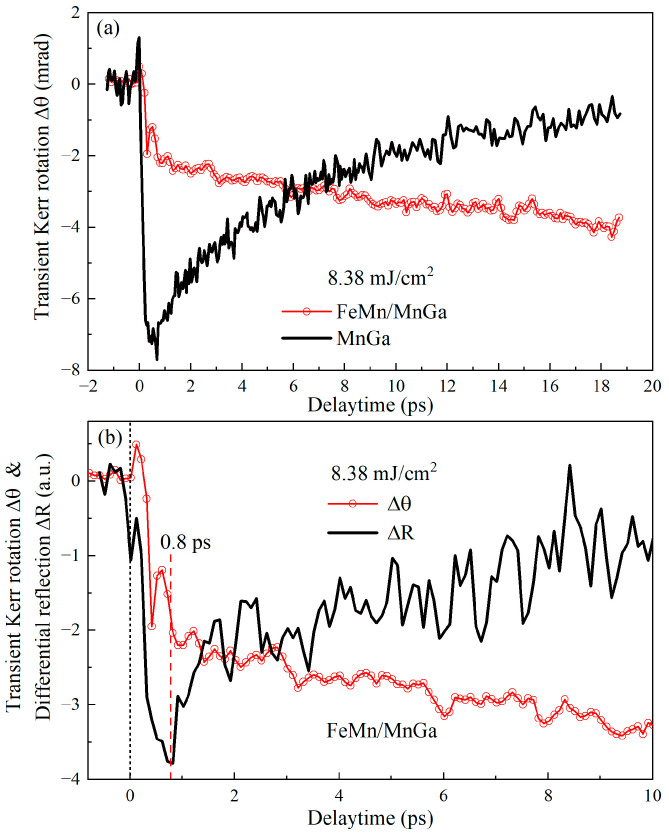
(**a**) The ultrafast demagnetization dynamics of FeMn/MnGa (open circles) and MnGa (solid line). (**b**) Short temporal traces of the Kerr signal (open circles) and transient reflectivity (solid line) of FeMn/MnGa upon photoexcitation.

To distinguish intralayer phonon–spin coupling interaction from interlayer phonon transport, a comparative experiment was carried out on the layered FeMn/MnGa sample and a single layer MnGa (12 nm) film. The measured ultrafast dynamics are plotted in Figure 3a for the pump fluence of 8.38 mJ/cm^2^. It is very exciting that two entirely different ultrafast dynamics of magnetization emerged. The ultrafast dynamics of laser-induced magnetization of a single layer MnGa film presented one-step ultrafast demagnetization in a subpicosecond time scale followed by a slower recovery of magnetization, whereas the bilayer FeMn/MnGa film presented two-step demagnetization: one fast demagnetization process in a subpicosecond time scale and one slower demagnetization process in several tens of picoseconds. The disappearance of the second slower process in the sole MnGa film reveals a weak or negligible intralayer phonon–spin coupling interaction. Consequently, the second-step slower demagnetization process may only originate from phonon-mediated interlayer coupling or phonon transport because electron and spin transports are ruled out due to their appearance in a time scale of less than one picosecond [15,17,19,20,22,30] and the extremely short diffusion length of electrons and spins in the FeMn layer [23,24,25].

To directly determine that the first-step fast demagnetization process is relevant to electrons, and to validate that the second-step slower demagnetization process is not directly relevant to electrons, we measured the transient reflectivity and Kerr signal of FeMn/MnGa bilayer film, as the solid line and open circles shown in Figure 3b, respectively. Transient reflectivity mainly reflects the relaxation dynamics of the temperature of excited electrons. It presented one-step ultrafast decay within 0.8 ps followed by a slower recovery or the temperature of excited electrons rose fast within 0.8 ps and then decayed slowly. Meanwhile, the first-step fast demagnetization also finished within 0.8 ps and agreed well with ultrafast demagnetization in the sole MnGa layer, as shown in Figure 3a. Consequently, we can assert that the first-step fast demagnetization in the FeMn/MnGa bilayer nanostructure comes from ultrafast electron–spin coupling in the sole MnGa layer. However, transient reflectivity does not contain a slower second-step decay process. As a result, the second-step slower demagnetization process is not directly relevant to the excited electrons. Therefore, we can definitively conclude that the second-step slower demagnetization originates from the phonon transport from FeMn into MnGa layers. Now, a clear physical picture can be drawn out. Photoexcited electrons transfer energy first into phonons in the FeMn and MnGa layers via electron–phonon coupling, and spins in MnGa layer via electron–spin coupling. The electron–spin coupling in the MnGa layer leads to the first-step subpicosecond ultrafast demagnetization. Then, phonon coupling between the FeMn and MnGa layers or phonon transport from the FeMn to the MnGa layers transfers further energy into the MnGa layer, which results in the emergence of the second-step slower demagnetization. However, we still do not know how phonon-mediated energy transfer from FeMn to MnGa layers reaches the spin subsystem in the MnGa layer, directly by phonon (FeMn)–spin (MnGa) coupling or indirectly by phonon (FeMn)–phonon (MnGa)–spin coupling? This will be further explored later.

### 3.1. Modeling of the Ultrafast Dynamics of Magnetization

To understand the ultrafast dynamics of demagnetization quantitatively, it is necessary to extract the time constants and amplitudes of the fast and slower demagnetization processes. A phenomenological model including two demagnetization processes has been developed, and written as:

(1) $$S_{k}\left(t\right)=C\left(t\right)⊗\left\{\theta \left(t\right)∙\left\{A_{f}\left[exp\left(-\frac{t}{{𝜏}_{f}}\right)-1\right]+A_{s}\left[exp\left(-\frac{t}{{𝜏}_{s}}\right)-1\right]\right\}\right\}$$

where the term in the first square bracket describes the dynamics of fast demagnetization with a time constant of 𝜏*_f_*, and a demagnetization amplitude of *A_f_*, while the term in the second square bracket denotes the dynamics of slow demagnetization with a time constant of 𝜏*_s_*, and a demagnetization amplitude of *A_s_*. *θ*(*t*) is a unit-step function, and *C*(*t*) represents the cross-correlation function of pump and probe pulses and can be approximated by a Gaussian function. Symbol ⊗ denotes the operation of the convolution.

**Figure 4:**
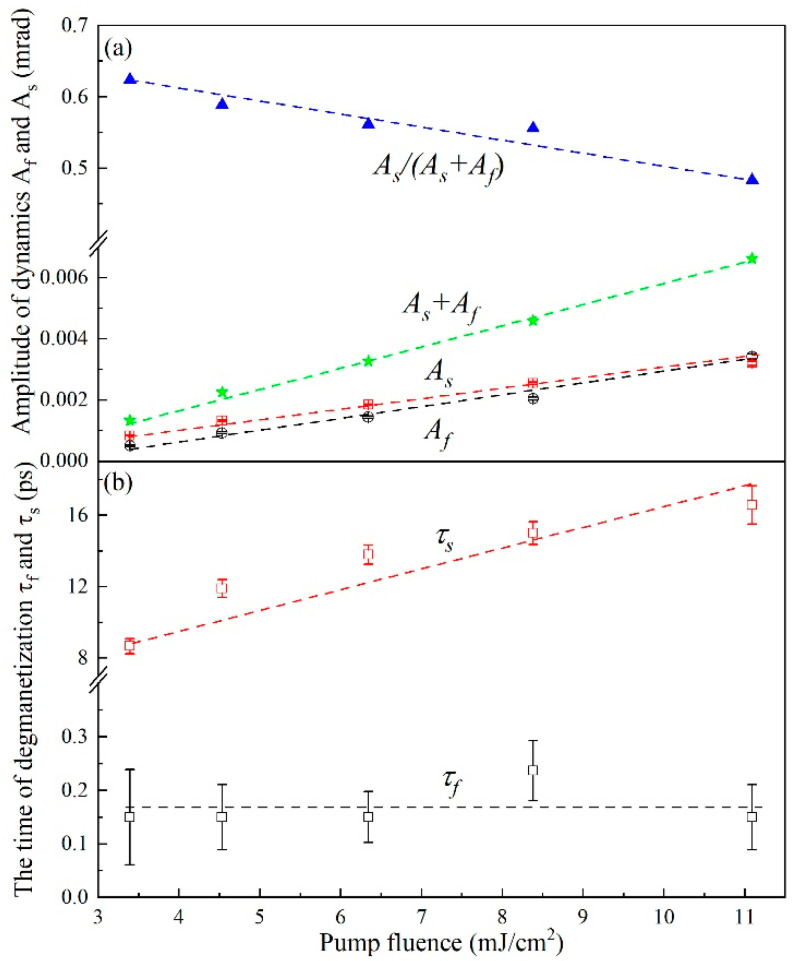
(**a**) The amplitudes of fast demagnetization (open circles, *A_f_*) and slow demagnetization (open squares, *A_s_*) versus the pump fluence. (**b**) The relaxation time 𝜏*_f_* and 𝜏*_s_* for laser-induced demagnetization are given as a function of the pump fluence. The dashed lines are the guiding eye.

The two-step demagnetization can be fitted well with Equation (1) using the Origin commercial software for scientific data analysis, as colored solid lines shown in Figure 2. The four extracted parameters, *A_f_*, *A_s_*, 𝜏*_f_* and 𝜏*_s_* as a function of pump fluence are plotted in Figure 4 by the scattered points. One can see that the time constant (𝜏*_f_*) of the fast demagnetization process is about 160 fs within experimental errors, and seems independent of pump fluence, as shown in Figure 4b, supporting the assertion that the mechanism of fast demagnetization originates from intrinsic spin flips [4,7]. However, the time constant of the slower demagnetization, 𝜏*_s_*, occurs at a timescale of 8–16 ps, increasing with pump fluence. Such an increase in 𝜏*_s_* with pump fluence agrees well with electron (FeMn)–phonon (FeMn)–couplings as the origin of the slower demagnetization because the higher pump fluence can generate a higher density of phonons that cause the enhancement of the re-absorption of phonons by electrons. Such an enhancement of the re-absorption of phonons slows down the process of electron-phonon thermal equilibrating in the FeMn layer due to the reverse flow of energy. Such a slowing of the electron-phonon thermal equilibrating process certainly causes the slowing down of subsequent phonon (FeMn)–mediated spin flips in the MnGa layer.

One can also see that *A_s_* is slightly stronger than *A_f_*. *A_s_* and *A_f_* increase almost linearly with pump fluence. Such an increase trend should be reasonable because the strength of the first-step fast demagnetization via electron–spin coupling is related to the excited electron density, whereas the strength of the second-step demagnetization via the interlayer phonon transport should also be almost linearly dependent on the excited electron density. On the other hand, it is notable that the proportion of the slower demagnetization in the total demagnetization varies with pump fluence. The ratio, *A_s_*/(*A_f_ + A_s_*), reduces from 0.624 to 0.483 with increasing pump fluence, which agrees well with the increase in 𝜏*_s_*. The increase in 𝜏*_s_* suggests more energy dissipated into environments or more energy losses which leads to the reduction in the increment of the second-step slower rise of the spin temperature.

### 3.2. Simulation Calculations Based on the Four-Temperature Model

To understand the microscopic mechanism of ultrafast two-step demagnetization, especially the exact energy transfer path in the second-step demagnetization, it was necessary to develop a model and to simulate the ultrafast dynamics of the two-step demagnetization quantitatively using this model. It has already been reported that the ultrafast dynamics of demagnetization in a single layer of ferromagnetic film could be well described by a 3T-M [1]. Here, an antiferromagnetic FeMn layer was added in our FeMn/MnGa bilayer film structure, and it contained two subsystems of electrons and phonons. In principle, an extended five-temperature model can describe well the ultrafast dynamics of demagnetization of our FeMn/MnGa bilayer film structure. However, the two electron subsystems, respectively, in the FeMn and MnGa layers, can be merged into one because the superdiffusive electron and spin transport between the FeMn and MnGa layers can be ignored due to the extremely short diffusion length of electrons and spins in FeMn. As a result, a four-temperature model (4T-M) is enough to describe the ultrafast dynamics of demagnetization in our bilayer structure. Based on 3T-M [1], our 4T-M can be written as:

$$C_{e}\left(T_{e}\right)\frac{{\mathrm{dT}}_{e}}{\mathrm{dt}}=-G_{\mathrm{es}}\left(T_{e}-{\;T}_{s}\right)-{\;G}_{\mathrm{el}}\left(T_{e}\;{-\;T}_{l}\right)+P\left(t\right)-{\;G}_{\mathrm{el}}^{\mathrm{FeMn}}\left(T_{e}-{\;T}_{l}^{\mathrm{FeMn}}\right)$$

$$\;\;\;{\;C}_{l}\frac{{\mathrm{dT}}_{l}}{\mathrm{dt}}=-{\;G}_{\mathrm{el}}\left(T_{l}-{\;T}_{e}\right)-{\;G}_{\mathrm{sl}}\left(T_{l}-{\;T}_{s}\right)-{\;G}_{\mathrm{ll}}\left(T_{l}-{\;T}_{l}^{\mathrm{FeMn}}\right)$$

$$C_{s}\frac{{\mathrm{dT}}_{s}}{\mathrm{dt}}=-{\;G}_{\mathrm{es}}\left(T_{s}-{\;T}_{e}\right)-\left(T_{s}-{\;T}_{l}\right)-{\;G}_{\mathrm{sl}}^{\mathrm{FeMn}}\left(T_{s}-{\;T}_{l}^{\mathrm{FeMn}}\right)$$

(2) $${\;C}_{l}^{\mathrm{MnFe}}\frac{{\mathrm{dT}}_{l}^{\mathrm{FeMn}}}{\mathrm{dt}}=-{\;G}_{\mathrm{el}}^{\mathrm{FeMn}}\left(T_{l}^{\mathrm{FeMn}}-{\;T}_{e}\right)-{\;G}_{\mathrm{sl}}^{\mathrm{FeMn}}\left(T_{l}^{\mathrm{FeMn}}-{\;T}_{s}\right)-{\;G}_{\mathrm{ll}}\left(T_{l}^{\mathrm{FeMn}\;}-{\;T}_{l}\right)$$

where *C_e_*, *C_s_*, *C_l_* and ${\;C}_{l}^{\mathrm{FeMn}}$ are the specific heats of the electrons, spins, lattice (phonon) subsystems of MnGa and lattice subsystem of FeMn, respectively, while *T_e_*, *T_s_*, *T_l_* and $T_{l}^{\mathrm{FeMn}}$ are their respective temperatures. *G_es_*, *G_el_*, *G_sl_*, $G_{\mathrm{el}}^{\mathrm{FeMn}}$ and $G_{\mathrm{sl}}^{\mathrm{FeMn}}$ are the coupling constants of the electron–spin, electron–lattice and spin–lattice interactions in the MnGa layer, the electron–lattice in the FeMn layer and the spin–lattice interactions between FeMn and MnGa layers, respectively. *G_ll_* is an effective phonon–phonon exchange coupling due to phonon transport. *P*(*t*) is the pump power absorbed by the electron subsystem per unit volume.

The first three equations in Equation (2) are just the modified 3T-M in the MnGa layer with the last term added in each equation to take account for the coupling interaction with phonons in the FeMn layer, while the fourth or last equation is newly added to describe the time evolution of the temperature of the phonon subsystem and the coupling interaction of phonons in the FeMn layer with the electron, spin and phonon subsystems in the MnGa layer. In simulation calculations, *P*(*t*) was approximated by the Gaussian form, *P*(*t*)= $\frac{P_{0}}{\sigma \sqrt{2\pi \;}}\exp\left(-\frac{t^{2}}{2\sigma^{2}}\right)$. *C_l_* = 1.83 × 10^6^ J(m^−3^K^−1^) and $C_{l}^{\mathrm{FeMn}}$ = 1.77 × 10^6^ J(m^−3^K^−1^) were set fixed based on the Debye law [32,33], while *C_e_*(*T_e_*) = *𝛾T_e_* and *𝛾* = 479 J(m^−3^K^−2^) were set [32,33]. The specific heat of the spins, *C_s_* = 2.32 × 10^5^ J(m^−3^K^−1^) in the MnGa film was calculated by *C_s_* = *C_tol_* − *C_e_*(*T* = 300 K) − *C_l_* [33,34], where *C_tol_* is ~2.2 × 10^6^ J(m^−3^K^−1^) [33]. The coupling constants between the electron and phonon subsystem were calculated by *G_el_*$=\frac{3\pi D_{F}^{2}D_{p}k_{B}^{2}T_{D}\lambda_{ep}^{2}}{2\hbar }$ [13,22], where *k_B_* is the Boltzmann constant, *T_D_* is the Debye temperature (*T_D_* = 275 K [33]; $T_{D}^{\mathrm{FeMn}}\;$= 560 K [32]), *D_F_* is the density of states at the Fermi level (*D_F_* = 1 eV^−1^atom^−1^ [33,35]; $D_{F}^{\mathrm{FeMn}}$ *=* 2 eV^−1^atom^−1^ [36], *D_P_* = 3 [22] is the number of oscillators per atomic site and *λ_ep_* is the electron–phonon coupling constant (*λ_ep_* = 0.04 eV [27,35]; $\lambda_{\mathrm{ep}}^{\mathrm{FeMn}}$ = 0.1 eV [37,38]). Consequently, *G_el_* and $G_{\mathrm{el}}^{\mathrm{FeMn}}$ were calculated as ~1.7 × 10^16^ W(m^−3^K^−1^) and ~9.4 × 10^17^ W(m^−3^K^−1^), respectively. *G_es_* = 1.7 × 10^17^ W(m^−3^K^−1^) was taken reasonably according to Ref. [1]. The phonon transport term or the interlayer phonon-phonon interaction, *G_ll_* can be written as *G_ll_* $=\frac{\;\xi_{p}}{L_{p}^{2}}$ (8.3 × 10^17^ W(m^−3^K^−1^)), where $\;\xi_{p}$= 30 W(m^−1^K^−1^) and *L_p_* = 6 nm was taken reasonably according to Refs. [31,39] for our sample. σ = 43 fs and P_0_ = 1 × 10^7^ mJ/cm^3^ were set. The remaining two parameters, *G_sl_* and $G_{\mathrm{sl}}^{\mathrm{FeMn}}$ were tuned until the ultrafast dynamics of two-step demagnetization were closely reproduced.

**Figure 5:**
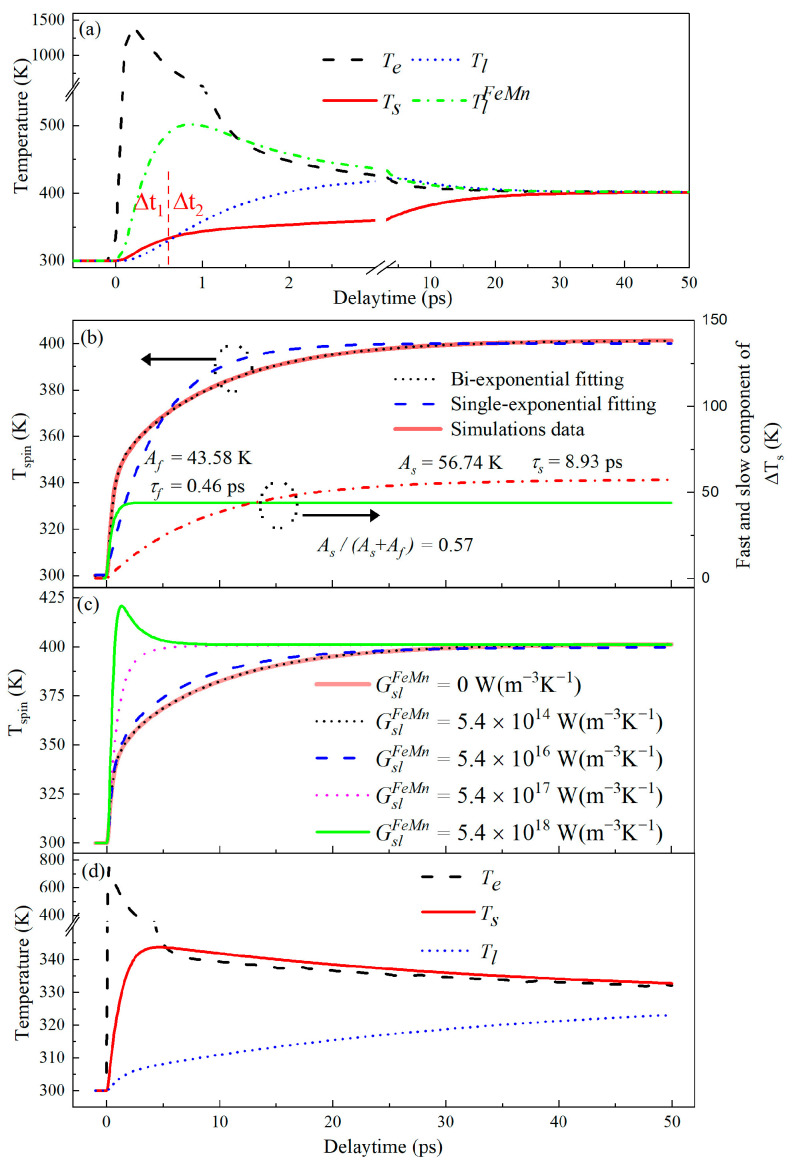
(**a**) Simulation temperature time evolution of electron (dash line), lattice of MnGa (dot line), lattice of FeMn (dot-dashed line), and spin (solid line) in an FeMn/MnGa layered structure at a pump fluence of 3.39 mJ/cm^2^ in a 4T-M model. The time window is divided into two regions Δt_1_ and Δt_2_ for convenient discussions. (**b**) The temperature in the spin system alone (red solid line). The blue dash and black dot lines are fits of a single- and double-exponential sum function in Equation (1) functions, respectively. (**c**) Simulation time evolution of the spin temperature in an FeMn/MnGa layered structure at various phonon (FeMn)-spin coupling coefficient ($G_{\mathrm{sl}}^{\mathrm{FeMn}}$) values in a 4T-M model. (**d**) Simulations of the time evolution of electron (dash line), lattice (dot line) and spin (solid line) temperatures in a sole MnGa layer by 4T-M as all coupling channels between the FeMn and MnGa layers are turned off.

Simulation calculations were performed based on 4T-M in Equation (2) for different values of *G_sl_* and $G_{\mathrm{sl}}^{\mathrm{FeMn}}$. It was found that the time evolution of *T*_s_ presented an ultrafast two-step rising process that corresponded to an ultrafast two-step demagnetization due to a linear correlation between *T*_s_ and magnetization when *G_sl_* was in order of 1.0 × 10^16^ W(m^−3^K^−1^) and $G_{\mathrm{sl}}^{\mathrm{FeMn}}$ was in order of 1.0 × 10^14^ W(m^−3^K^−1^). At this moment, by adjusting *G_sl_* and $G_{\mathrm{sl}}^{\mathrm{FeMn}}$ carefully, the ultrafast dynamics of two-step demagnetization were reproduced well as *G_sl_* =1.5 × 10^16^ W(m^−3^K^−1^) and $G_{\mathrm{sl}}^{\mathrm{FeMn}}$ ≤ 5.4 × 10^14^ W(m^−3^K^−1^). Simulating results are plotted in Figure 5a and Figure 5b, respectively, for the FeMn/MnGa bilayer and single MnGa films. One can see clearly the time evolution of the temperatures of the four subsystems of electrons, spins, phonons in MnGa and phonons in FeMn from Figure 5a. The temperature of the electron subsystem, *T*_e_, first rises sharply in 200 fs after pump excitation, as the black dashed line shows. Meanwhile, the temperature of the spin subsystem, *T_s_*, also performs the first-step fast rising within Δt_1_ = ~600 fs, as the red line shows in the Δt_1_ window, while the temperature of the phonon subsystem in the FeMn layer, $T_{l}^{\mathrm{FeMn}}$, also rises sharply up to a peak temperature in the Δt_1_ interval via the strong electron–phonon coupling, as the green dash-dot line shows. *T_l_* is smaller than *T_s_* in the Δt_1_ window, implying that the first-step demagnetization is possibly driven via intralayer phonons, but only possibly via electrons and interlayer phonons because of *T_e_* > *T_s_*, and $T_{l}^{\mathrm{FeMn}}$ > *T_s_*. However, good simulations were obtained under the condition of $G_{\mathrm{sl}}^{\mathrm{FeMn}}$ ≤ 5.4 × 10^14^ W(m^−3^K^−1^). So a small $G_{\mathrm{sl}}^{\mathrm{FeMn}}$ ≤ 5.4 × 10^14^ W(m^−3^K^−1^) implies that the interlayer phonon–spin coupling channel is closed. Consequently, the first-step fast demagnetization can only come from electron–spin coupling, agreeing well with the experimental results in Figure 3. After the Δt_1_ interval, *T_l_* and *T_s_* keep increasing slowly, but *T_l_* increases faster than *T_s_* and becomes obviously higher than *T_s_*, leading to an energy transfer from phonons in the MnGa layer to spin. Therefore, this simulation revealed that the second-step slow demagnetization originates from phonon (FeMn)–phonon (MnGa)–spin coupling. To clearly show the two-step process of *T_s_* rising, *T_s_* is alone plotted in Figure 5b. Its fit with the single exponential and double-exponential sums in Equation (1) functions are also plotted in Figure 5b by blue dash and black dot lines, respectively. The double-exponential sum function fits the simulation data very well, but the single exponential function fails to fit well. The good double-exponential fit gives the time constants of fast and slow processes as *𝜏_f_* = ~0.46 ps and *𝜏_s_* = ~8.39 ps, respectively, while the ratio of the amplitude (*A*_s_) of the slow component to the total increment (*A_f_* + *A_s_*), *A_s_*/(*A_f_* + *A_s_*) reaches ~0.56. These parameters agree well with those in Figure 4 at the pump fluence of 3.39 mJ/cm^2^.

To intuitively reveal the origin of the second-step slow demagnetization, we studied the effect of the magnitude of the phonon (FeMn)–spin coupling coefficient ($G_{\mathrm{sl}}^{\mathrm{FeMn}}$) on the second-step slow demagnetization process or the second-step slow rising process of *T*_s_. As the blue dash line shows in Figure 5c, the second-step slow rising accelerates as $G_{\mathrm{sl}}^{\mathrm{FeMn}}$ increases to 5.4 × 10^16^ W(m^−3^K^−1^) which implies the phonon (FeMn)–spin (MnGa) coupling channel is opened. It disappears and the first-step fast process enhances as $G_{\mathrm{sl}}^{\mathrm{FeMn}}$ increases to 5.4 × 10^17^ W(m^−3^K^−1^) and above, as the magenta dot-dashed and green lines show, respectively. The enhancement of $G_{\mathrm{sl}}^{\mathrm{FeMn}}$ means the weakening of energy transfer via the phonon (FeMn)–phonon (MnGa)–spin coupling channel, leading to the disappearance of the second-step slow rising process of *T*_s_. This intuitively shows that the second-step slow demagnetization process originates from phonon transport via the phonon (FeMn)–phonon (MnGa)–spin coupling channel, rather than from phonon (FeMn)–spin coupling.

We also carried out the simulation calculations of ultrafast demagnetization dynamics in a single layer of MnGa by turning off all the coupling channels between the FeMn and MnGa layers by setting $G_{\mathrm{el}}^{\mathrm{FeMn}}=0$, *G_ll_* = 0 and $G_{\mathrm{sl}}^{\mathrm{FeMn}}=0$, and maintaining all the other parameters consistent with those used in Figure 5a except for reducing *P*_0_ to deduct the absorbed energy of the FeMn layer. The simulation results are plotted in Figure 5d. *T_s_* obviously presents the ultrafast dynamics of one-step demagnetization, as the red line shows, agreeing well with experimentally measured one-step ultrafast demagnetization in a single MnGa layer as shown in Figure 3a. Such a good agreement shows the validity of our 4T-M and the rationalities of all parameters used in the calculations.

## 4. Conclusions

An antiferromagnet/ferromagnet bilayer nanostructure, FeMn/MnGa film, was designed and grown on a GaAs substrate by MBE. MBE growth guaranteed the consistency of the lattice structure in the FeMn and MnGa layers. In other words, almost no phonon scattering occurred at the magnetic interface of the FeMn/MnGa heterostructure so that phonons could transport easily from the FeMn into the MnGa layers. An FeMn antiferromagnetic layer was selected to suppress possible electron and spin transports because of the extremely short diffusion length of electrons and spins in the antiferromagnetic materials. The ultrafast dynamics of demagnetization were studied on the FeMn/MnGa bilayer and MnGa single layer films comparatively with TR-MOKE spectroscopy. The ultrafast dynamics of two-step demagnetizations, including the first-step fast and the second-step slow demagnetizations, were observed on the FeMn/MnGa film, whereas only the ultrafast dynamics of the first-step fast demagnetization occurred on the single MnGa film. These comparative results reveal that the first-step fast demagnetization originates from the sole MnGa layer, while the second-step slow demagnetization comes from phonon transport because possible electron and spin transports are suppressed in FeMn antiferromagnetic films, and they should occur in a subpicosecond time scale. Transient reflectivity reveals that the ultrafast rising process of the temperature of excited electrons agrees well with the first-step fast demagnetization, implying that the first-step fast demagnetization originates from electron–spin coupling. To understand the exact path of FeMn-phonon-mediation of the second-step slow demagnetization, we developed a four temperature model considering phonon transport on the basis of 3T-M. Simulation calculations were performed based on this 4T-M, and reproduced well the ultrafast dynamics of two-step demagnetization observed as the phonon (FeMn)–spin (MnGa) coupling channel was closed. However, the second-step slow demagnetization accelerated until it became fast and occurred in a subpicosecond time scale as the phonon (FeMn)–spin (MnGa) coupling channel opened and its coupling strength increased. This indicates that the second-step slow demagnetization originates from FeMn phonon–phonon (MnGa)–spin coupling. In other words, we observed ultrafast demagnetization driven by phonon transport in an FeMn/MnGa bilayer structure.

## Data Availability

The data presented in this study are available on request from the corresponding author.
